# Epidemiology and Outcome of Pneumonia Caused by Methicillin-Resistant *Staphylococcus aureus* (MRSA) in Canadian Hospitals

**DOI:** 10.1371/journal.pone.0075171

**Published:** 2013-09-17

**Authors:** Manal Tadros, Victoria Williams, Brenda L. Coleman, Allison J. McGeer, Shariq Haider, Christine Lee, Harris Iacovides, Ethan Rubinstein, Michael John, Lynn Johnston, Shelly McNeil, Kevin Katz, Nancy Laffin, Kathryn N. Suh, Jeff Powis, Stephanie Smith, Geoff Taylor, Christine Watt, Andrew E. Simor

**Affiliations:** 1 University of Toronto, Toronto, Ontario, Canada; 2 Sunnybrook Health Sciences Centre, Toronto, Ontario, Canada; 3 Mount Sinai Hospital, Toronto, Ontario, Canada; 4 Hamilton Health Sciences Centre, Hamilton, Ontario, Canada; 5 St. Joseph’s Health Care, Hamilton, Ontario, Canada; 6 Health Sciences Centre, Winnipeg, Manitoba, Canada; 7 London Health Sciences Centre, London, Ontario, Canada; 8 Queen Elizabeth II Health Sciences Centre, Halifax, Nova Scotia, Canada; 9 North York General Hospital, Toronto, Ontario, Canada; 10 The Ottawa Hospital, Ottawa, Ontario, Canada; 11 Toronto East General Hospital, Toronto, Ontario, Canada; 12 University of Alberta Hospital, Edmonton, Alberta, Canada; Amphia Ziekenhuis, Netherlands

## Abstract

**Background:**

MRSA remains a leading cause of hospital-acquired (HAP) and healthcare-associated pneumonia (HCAP). We describe the epidemiology and outcome of MRSA pneumonia in Canadian hospitals, and identify factors contributing to mortality.

**Methods:**

Prospective surveillance for MRSA pneumonia in adults was done for one year (2011) in 11 Canadian hospitals. Standard criteria for MRSA HAP, HCAP, ventilator-associated pneumonia (VAP), and community-acquired pneumonia (CAP) were used to identify cases. MRSA isolates underwent antimicrobial susceptibility testing, and were characterized by pulsed-field gel electrophoresis (PFGE) and Panton-Valentine leukocidin (PVL) gene detection. The primary outcome was all-cause mortality at 30 days. A multivariable analysis was done to examine the association between various host and microbial factors and mortality.

**Results:**

A total of 161 patients with MRSA pneumonia were identified: 90 (56%) with HAP, 26 (16%) HCAP, and 45 (28%) CAP; 23 (14%) patients had VAP. The mean (± SD) incidence of MRSA HAP was 0.32 (± 0.26) per 10,000 patient-days, and of MRSA VAP was 0.30 (± 0.5) per 1,000 ventilator-days. The 30-day all-cause mortality was 28.0%. In multivariable analysis, variables associated with mortality were the presence of multiorgan failure (OR 8.1; 95% CI 2.5-26.0), and infection with an isolate with reduced susceptibility to vancomycin (OR 2.5, 95% CI 1.0-6.3).

**Conclusions:**

MRSA pneumonia is associated with significant mortality. Severity of disease at presentation, and infection caused by an isolate with elevated MIC to vancomcyin are associated with increased mortality. Additional studies are required to better understand the impact of host and microbial variables on outcome.

## Introduction

Methicillin-resistant *Staphylococcus aureus* (MRSA) is among the most frequently identified pathogens causing healthcare-associated pneumonia (HCAP), hospital-acquired pneumonia (HAP), and ventilator-associated pneumonia (VAP) worldwide [1,2]. Although it appears to be a relatively uncommon cause of community-acquired pneumonia (CAP) [3], MRSA may be an important emerging respiratory pathogen in the community, associated with severe necrotizing infection especially following recent influenza [4-6]. Regardless of the setting, MRSA pneumonia is associated with significant morbidity and mortality [7,8]. However, the epidemiology of MRSA pneumonia is not well understood, and in particular, the relative importance of host and microbial factors in affecting its outcome is uncertain. Host factors such as underlying comorbidities or measures of severity of illness have been associated with poor outcome [9,10]. Other studies examining the role of putative microbial virulence factors such as the presence of the Panton-Valentine leukocidin (PVL) gene or of reduced susceptibility to glycopeptide antimicrobial agents, have yielded conflicting results [9,11-15]. The objectives of this study were to describe the epidemiology and outcomes associated with MRSA pneumonia in adults in Canadian hospitals, and to identify host and microbial variables associated with mortality.

## Materials and Methods

### Ethics statement

The study was approved by the Research Ethics Board, Sunnybrook Research Institute, Sunnybrook Health Sciences Centre (Study No. 356-2009). All data submitted to the study investigators were anonymized, and no personal identifying information was available to the investigators. The study was observational laboratory-based surveillance with review of medical records. Study investigators and research associates had no direct patient contact and the study protocol did not involve any change in patient care or management; all decisions regarding patient investigation and treatment were at the discretion of the attending physician. The Research Ethics Board approved this study without any requirement for patient consent (neither verbal nor written).

### Study design

Prospective surveillance for MRSA pneumonia in adults (≥ 18 years of age) was conducted in 11 Canadian hospitals from January 1 to December 31, 2011. Participating hospitals were in four Canadian provinces, and had a mean of 610 beds (range: 404-1,500 beds); nine were tertiary-care teaching hospitals. Surveillance was laboratory-based. Whenever MRSA was recovered in culture from a respiratory specimen (sputum, endotracheal aspirate, bronchoscopy specimen, lung biopsy, or pleural fluid), or a blood culture the patient’s medical records were reviewed by experienced infection prevention and control personnel to determine whether criteria for MRSA pneumonia were present. Patients could not be included in this study more than once.

### Study definitions

The diagnosis of pneumonia required clinical features (fever or hypothermia, new or increasing cough or dyspnea, production of purulent sputum, or leukocytosis) in association with new or progressive pulmonary infiltrates suggestive of infection on chest x-ray [16]. MRSA was considered to be the etiologic agent if it was cultured from pleural fluid or a protected bronchoscopy specimen, or from another respiratory specimen in association with a Gram stain that revealed ≥ 25 white blood cells per high power field and concordant bacterial morphology. If the organism was recovered only from a blood culture, a diagnosis of MRSA pneumonia was accepted if criteria for pneumonia were met, and the bacteremia was not associated with another site of infection. Patients with infective endocarditis were excluded.

HCAP, HAP, and VAP were defined using previously published criteria [1,17]. CAP was defined as lower respiratory infection with onset less than 48 hours after hospital admission and no other hospitalization in the previous 90 days or other significant healthcare exposure in the prior 30 days. Place of MRSA acquisition (healthcare facility or community) was ascertained based on available epidemiologic data and using previously published criteria [18].

Necrotizing pneumonia was determined to be present based on typical radiographic features detected on a contrast-enhanced chest CT scan [19]. Multiorgan failure was defined as hypotension in association with at least two of acute renal injury, acute hepatic injury, or coagulopathy. Empiric antimicrobial therapy was defined as treatment prescribed within the first 48 hours after a diagnosis of pneumonia was made, but before MRSA had been identified in culture. Appropriate antimicrobial therapy included treatment with vancomycin or linezolid; treatment with clindamycin was also considered to be appropriate if the isolate was susceptible *in vitro* [20].

### Data collection

The medical records of all eligible patients were reviewed for demographic, clinical and epidemiologic data. If treatment included vancomycin, the initial trough vancomycin levels were noted. Outcome (all-cause mortality) and complications related to the pneumonia were determined at 30 days of follow-up from the diagnosis of infection.

### Laboratory methods

The initial MRSA isolate from each patient was characterized in a central microbiology laboratory. MRSA was confirmed by detection of the *nuc* and *mecA* genes by polymerase chain reaction (PCR) [21]. Antimicrobial susceptibility testing was done by broth microdilution in accordance with Clinical and Laboratory Standards Institute guidelines [22]. Inducible clindamycin resistance was detected using the D-test disk diffusion method [22]. Vancomycin minimum inhibitory concentrations (MICs) were determined using the Etest (AB bioMérieux, Solna, Sweden).

Molecular typing of isolates was done by pulsed-field gel electrophoresis (PFGE) following DNA extraction and digestion with *Sma*I. PFGE-generated profiles were digitized and analyzed using BioNumerics software, version 5.10 (Applied Maths) [18,23]. Strains designated as PFGE CMRSA-10 resemble the community-associated (CA-MRSA) genotype USA300 (multilocus sequence type 8 [ST8]; clonal complex 8 [CC8]); CMRSA-2 resembles USA100/800 (ST5; CC5). The presence of the PVL gene was determined by PCR [24].

### Statistical methods

The incidence of MRSA pneumonia was calculated as the number of cases per 1,000 admissions and per 10,000 patient-days. VAP incidence was calculated as the number of cases per 1,000 ventilator-days. To assess differences between patient populations, categorical variables were expressed as proportions and were compared using the chi-square test or the Fisher exact test as appropriate. Continuous variables were compared using Student’s *t* test. All tests were 2-tailed, and a *P* value < 0.05 was considered to be statistically significant. Logistic regression was done to evaluate the association between outcome (30-day all-cause mortality) and certain host- or microbial-related variables. Variables considered in the model were selected *a priori* based on previously published data [9,13,25], and included age (as a continuous variable), presence of underlying comorbidities (chronic pulmonary, cardiac, or renal disease), pneumonia acquisition (CAP or HCAP/HAP), presence of MRSA bacteremia or multiorgan failure, use of appropriate empiric antimicrobial therapy (prior to identification of MRSA), infecting strain of MRSA with the PVL gene, or with reduced susceptibility to vancomcyin. The goodness of fit of the final model was tested using the goodness-of-fit chi-squared test. All analyses were done using SPSS, Version 16.0 (SPSS Inc., Chicago IL).

## Results

A total of 161 patients with MRSA pneumonia were identified: 90 (55.9%) had HAP, 26 (16.1%) had HCAP, and 45 (28.0%) had CAP. Thirty-eight (42.2%) of the nosocomial pneumonias were acquired in an intensive care unit (ICU), and 23 patients (14.3%) had VAP. The mean (± SD) incidence of MRSA pneumonia was 0.34 (± 0.17) per 1,000 admissions (range: 0.14-0.65/1,000 admissions). The mean incidence of MRSA HAP was 0.32 (± 0.26) cases per 10,000 patient-days (range: 0.11-0.78/10,000 patient-days), and of MRSA VAP was 0.30 (± 0.5) per 1,000 ventilator-days (range: 0-1.76/1,000 ventilator-days).

Most (67.1%) patients with MRSA pneumonia were male, and the mean age was 64.2 (± 17.8) years (median age, 68 yrs). Almost all patients (95.0%) had at least one underlying comorbidity, most commonly cardiac disease (31.7%), chronic pulmonary disease (29.2%), or diabetes mellitus (28.0%) (Table 1). Only two (1.2%) patients had prior laboratory-confirmed influenza virus infection. Approximately half (50.9%) of the patients were previously known to have been colonized with MRSA. The initial respiratory specimen that yielded MRSA in patients with pneumonia was sputum in 38%, endotracheal aspirate (28%), and bronchoalveolar lavage (14%); protected bronchoalveolar lavage or brushings were infrequently available. Blood cultures from 31 (19%) patients yielded MRSA, and two (1.2%) patients had MRSA recovered from pleural fluid specimens. MRSA was the only respiratory pathogen identified in most (77%) patients.

**Table 1:** Characteristics of patients with healthcare-associated (HCAP), hospital-acquired (HAP), and community-acquired (CAP) methicillin-resistant *Staphylococcus aureus* (MRSA) pneumonia.

	**No. (%**)** with the characteristic**			
**Variable**	**All (n=161**)	**HCAP/HAP (n=116**)	**CAP (n=45**)	***P* value**
Mean age (± SD), yrs	64.2 ± 17.8	68.4 ± 14.7	53.5 ± 20.5	< 0.001
No. (%) male	108 (67.1)	76 (65.5)	32 (67.1)	0.577
Underlying comorbidities				
None	8 (5.0)	3 (2.6)	5 (11.1)	0.039
Cardiac disease	51 (31.7)	43 (37.1)	8 (17.8)	0.023
Chronic pulmonary disease	47 (29.2)	34 (29.3)	13 (28.9)	0.992
Diabetes mellitus	45 (28.0)	36 (31.0)	9 (20.0)	0.177
Renal disease	19 (11.8)	18 (15.5)	1 (2.2)	0.026
Cirrhosis or chronic hepatitis	7 (4.3)	7 (6.0)	0	0.192
Chemotherapy/radiotherapy	7 (4.3)	6 (5.1)	1 (2.2)	0.671
Neutropenia	4 (2.5)	2 (1.7)	2 (4.4)	0.311
HIV infection	3 (1.9)	0	3 (6.7)	0.021
Prior laboratory-confirmed influenza	2 (1.2)	2 (1.7)	0	0.991
Prior known MRSA colonization	82 (50.9)	62 (53.4)	20 (44.4)	0.383
MRSA acquisition				
Nosocomial/healthcare-associated	139 (86.3)	111 (95.7)	28 (62.2)	< 0.001
Community	22 (13.7)	5 (4.3)	17 (37.8)	
Complications				
MRSA bacteremia	38 (23.6)	24 (20.7)	14 (31.1)	0.153
Admitted to ICU (% of those not already in ICU)	39 (31.7)	21 (26.9)	18 (40.0)	0.538
Necrotizing pneumonia/empyema/lung abscess	13 (8.1)	6 (5.2)	7 (15.6)	0.049
Multiorgan failure	21 (13.0)	17 (14.7)	4 (8.9)	0.438
All-cause 30-day mortality	45 (28.0)	36 (31.0)	9 (20.0)	0.177
Death within 48 hrs of onset of pneumonia	10 (6.2)	8 (6.9)	2 (4.4)	0.727
Management				
Infectious diseases consultation	66 (41.0)	45 (38.8)	21 (46.7)	0.350
Appropriate empiric antimicrobial therapy^a^	52 (32.2)	36 (31.0)	16 (35.6)	0.579
Mean (± SD) days of antimicrobial therapy	14.0 (± 13.0)	14.3 (± 14.0)	13.2 (± 10.2)	0.651
Mean (± SD) vancomycin trough level (µg/mL)^b^	14.4 (± 9.4)	15.3 (± 10.4)	12.1 (± 6.4)	0.125
MRSA PFGE type ^c^				
CMRSA-2 (USA100/800)	83 (57.2)	73 (70.9)	10 (23.8)	< 0.001
CMRSA-10 (USA300)	40 (27.6)	20 (19.4)	20 (47.6)	
Other types	22 (15.2)	10 (9.7)	12 (28.6)	
PVL gene present	41 (28.3)	19 (18.4)	22 (52.4)	< 0.001
Vancomycin MIC (µg/mL)^d^				
≤ 0.5	7 (4.8)	5 (4.8)	2 (4.9)	
1.0	58 (40.0)	39 (37.9)	19 (45.2)	
1.5	73 (50.3)	52 (50.5)	21 (50.0)	
2.0	7 (4.8)	7 (6.8)	0	0.102

a Appropriate empiric antimicrobial therapy, treatment with vancomycin, linezolid, or clindamycin if the MRSA isolate was susceptible, in the 24-48 hrs prior to the availability of culture results.

b Vancomycin trough levels, among those treated with vancomycin and for whom vancomycin levels were available.

c PFGE, pulsed-field gel electrophoresis (145 isolates available for molecular typing).

d Vancomycin MIC, minimal inhibitory concentration (µg/mL) as determined by Etest (145 isolates available for susceptibility testing).

As compared to patients with MRSA HCAP or HAP, patients with MRSA CAP were younger (mean age 53.5 ± 20.5 yrs vs 68.4 yrs ± 14.7 yrs respectively; *P* < 0.001), and more likely to be without underlying comorbidities (11.1% vs 2.6% respectively; Odds Ratio [OR] 4.72, 95% Confidence Interval [CI] 1.08-20.41; *P* = 0.039) (Table 1). MRSA CAP was more likely to have been associated with the development of necrotizing pneumonia, empyema, or lung abscess, although only 10 (6.2%) patients had these complications.

Only 52 (32.2%) patients received empiric antimicrobial therapy with an agent to which MRSA was susceptible *in vitro* (50 patients treated with vancomycin, one patient with linezolid, and one with clindamycin). Eventually, 133 (82.6%) patients were treated with vancomycin, and 15 (9.3%) received linezolid. The mean (±SD) duration of antimicrobial therapy was 13.3 (± 10.6) days. Of those treated with vancomycin, trough levels were measured at least once and the results were available for 97 (72.9%) patients; the mean (±SD) initial trough vancomycin level was 14.3 (± 9.4) mg/L.

There were 145 (90.0%) MRSA isolates available for laboratory characterization. Of these, 57.2% were healthcare-associated strains (CMRSA-2/USA100/800) based on PFGE typing, and 27.6% were a CA-MRSA clone (CMRSA-10/USA300). Isolates associated with CAP were more likely to be CMRSA-10/USA300 (47.6% vs 19.4% respectively; OR 7.30, 95% CI 2.95-18.06; *P*<0.001) and to possess the PVL gene (52.4% vs 18.4% respectively; OR 4.86, 95% CI 2.22-10.65; *P*<0.001) (Table 1). There were 41 (28.3%) isolates that had the PVL gene, and these strains were more likely to be recovered from younger patients, from those without underlying comorbidities, and from those with CAP (Table 2). Infection with a PVL-positive isolate was also more likely to be complicated by necrotizing pneumonia, empyema, or lung abscess (17.1% vs 4.8% respectively; OR 4.08, 95% CI 1.21-13.70 *P* = 0.038), but there was no difference in the 30-day mortality in those patients who were infected by strains with or without the PVL gene (26.8% vs 28.8% respectively; *P* = 0.96). The distribution of vancomycin MICs as determined by Etest is summarized in Table 1. Resistance to vancomycin was not detected, and only 7 (4.8%) had a vancomycin MIC of 2.0 µg/mL. Most isolates were resistant to clindamycin and erythromycin (68.3% and 91.0%, respectively), but the majority were susceptible to cotrimoxazole (95.5%), tetracycline (92.5%), and tigecycline (97.9%). All isolates were susceptible to linezolid.

**Table 2:** Characteristics of patients with methicillin-resistant *Staphylococcus aureus* (MRSA) pneumonia associated with the presence or absence of the Panton-Valentine Leukocidin (PVL) gene.

**Variable**	**No. (%**)		**Odds Ratio (95% CI**)	***P* value**
	**PVL gene present (n=41**)	**PVL gene absent (n=104**)		
Mean age (± SD), yrs	55.2 ± 16.8	67.9 ± 16.0		< 0.001
No. (%) male	28 (68.3)	68 (65.4)	1.14 (0.53-2.47)	0.577
Underlying comorbidities				
None	4 (9.8)	2 (1.9)	1.82 (0.83-1.93)	0.054
Cardiac disease	7 (17.1)	39 (37.5)	0.34 (0.14-0.85)	0.018
Chronic pulmonary disease	14 (34.1)	30 (28.8)	1.28 (0.59-2.77)	0.552
Diabetes mellitus	5 (12.2)	31 (29.8)	0.33 (0.12-0.91)	0.033
Renal disease	1 (2.4)	15 (14.4)	0.15 (0.02-1.02)	0.041
Cirrhosis or chronic hepatitis	0	6 (5.8)	Undefined	0.184
Neutropenia	3 (7.3)	0	Undefined	0.021
HIV infection	2 (4.9)	1 (1.0)	5.28 (0.47-59.91)	0.193
Prior laboratory-confirmed influenza	1 (2.4)	1 (1.0)	2.58 (0.16-42.17)	0.487
Prior known MRSA colonization	17 (41.5)	56 (53.8)	0.61 (0.29-1.26)	0.200
MRSA acquisition				
Nosocomial/healthcare-associated	29 (70.7)	95 (91.3)		
Community	12 (29.3)	9 (8.7)	4.37 (1.67-11.40)	0.003
MRSA pneumonia				
HCAP/HAP^a^	19 (46.3)	84 (80.8)		
CAP^a^	22 (53.7)	24 (19.2)	4.86 (2.22-10.65)	< 0.001
Complications				
MRSA bacteremia	14 (34.1)	20 (19.6)	2.13 (0.95-4.78)	0.082
Admitted to ICU (% of those not already in ICU)	15 (48.4)	22 (36.7)	1.62 (0.67-3.90)	0.368
Necrotizing pneumonia/empyema/lung abscess	7 (17.1)	5 (4.8)	4.08 (1.21-13.70)	0.038
Multiorgan failure	5 (12.2)	13 (12.5)	0.97 (0.32-2.92)	0.998
All-cause 30-day mortality	11 (26.8)	30 (28.8)	0.90 (0.40-2.04)	0.987
Death within 48 hrs of onset of pneumonia	3 (7.3)	7 (6.7)	1.09 (0.27-4.45)	0.900
Management				
Infectious diseases consultation	21 (51.2)	38 (37.6)	1.74 (0.84-3.62)	0.188
Appropriate empiric antimicrobial therapy^b^	17 (41.5)	28 (26.9)	1.92 (0.81-4.12)	0.111
Mean (± SD) days of antimicrobial therapy	11.9 (± 10.0)	15.1 (± 14.5)		0.209
Mean (± SD) vancomycin trough level (µg/mL)^c^	9.7 (± 6.6)	15.7 (± 9.9)		0.008
Vancomycin trough level (µg/mL)^c^				
< 15.0	17 (73.9)	36 (53.7)		
≥ 15.0	6 (26.1)	31 (46.3)	0.41 (0.14-1.17)	0.14
MRSA PFGE type ^d^				
CMRSA-2 (USA100/800)	0	83 (79.8)		
CMRSA-10 (USA300)	36 (87.8)	4 (3.8)	18.9 (7.99-44.74)	< 0.001
Other types	5 (12.2)	17 (16.4)		
Vancomycin MIC (µg/mL)^e^				
≤ 1.0	28 (68.3)	37 (35.6)		
≥ 1.5	13 (31.7)	67 (64.4)	0.26 (0.12-0.55)	< 0.001

a HCAP, healthcare-associated pneumonia; HAP, hospital-acquired pneumonia; CAP, community-acquired pneumonia

b Appropriate empiric therapy, treatment with vancomycin, linezolid, or clindamycin if the MRSA isolate was susceptible, in the 24-48 hrs prior to the availability of culture results.

c Vancomycin trough levels, among those treated with vancomycin and for whom vancomycin levels were available.

d PFGE, pulsed-field gel electrophoresis (145 isolates available for molecular typing).

e Vancomycin MIC, minimal inhibitory concentration (µg/mL) as determined by Etest (145 isolates available for susceptibility testing).

MRSA bacteremia was detected in 38 (23.6%) patients, and 21 (13.0%) had evidence of multiorgan failure within 24 hours of the onset of the pneumonia. Of those who were not already in an ICU at the time MRSA pneumonia was identified, 39 (31.7%) were subsequently admitted or transferred to an ICU. A total of 45 patients died (all-cause 30-day mortality, 28.0%); 10 (6.2%) patients died within 48 hours of the onset of the infection. The results of a univariate analysis of variables associated with 30-day mortality are summarized in Table 3. Mortality was higher in patients with underlying cirrhosis or chronic hepatitis, MRSA bacteremia, or multiorgan failure. Infection caused by isolates with vancomycin MIC ≥ 1.5 µg/mL was also associated with increased risk of dying, but vancomycin trough levels ≥ 15 µg/mL were not associated with improved outcome.

**Table 3:** Variables associated with 30-day all-cause mortality in univariate analysis in patients with methicillin-resistant *Staphylococcus aureus* (MRSA) pneumonia.

**Variable**	**No. (%**)		**Odds Ratio (95% CI**)	***P* value**
	**Alive at 30 days (n=116**)	**Dead at 30 days (n=45**)		
Mean age (± SD), yrs	63.0 (18.5)	67.4 (15.5)		0.152
No. (%) male	78 (67.2)	30 (66.7)	0.97 (0.47-2.02)	0.998
Underlying comorbidities				
None	7 (6.0)	1 (2.22)	0.48 (0.34-23.64)	0.444
Cardiac disease	36 (26.7)	15 (33.3)	1.11 (0.53-2.32)	0.851
Chronic pulmonary disease	30 (25.9)	17 (37.8)	1.74 (0.84-3.62)	0.176
Diabetes mellitus	31 (26.7)	14 (31.1)	1.24 (0.58-2.63)	0.565
Renal disease	11 (9.5)	8 (17.8)	2.06 (0.77-5.26)	0.174
Cirrhosis or chronic hepatitis	2 (1.7)	5 (11.1)	7.13 (1.33-38.19)	0.019
Neutropenia	1 (0.9)	3 (6.7)	8.21 (0.83-81.16)	0.067
HIV infection	2 (1.7)	1 (2.2)	1.30 (0.12-14.65)	1.00
Prior laboratory-confirmed influenza	2 (1.7)	0	0.98 (0.96-1.01)	1.00
Prior known MRSA colonization	56 (48.3)	26 (57.8)	1.47 (0.73-2.94)	0.30
MRSA acquisition				
Nosocomial/healthcare-associated	99 (85.3)	40 (88.9)		
Community	17 (14.7)	5 (11.1)	0.73 (0.25-2.11)	0.621
Pneumonia acquisition				
HCAP/HAP^a^	80 (69.0)	36 (80.0)		
CAP^a^	36 (31.0)	9 (20.0)	0.56 (0.24-1.27)	0.177
Complications				
MRSA bacteremia	20 (17.2)	18 (41.9)	3.46 (1.59-7.49)	0.003
Multiorgan failure	5 (4.3)	16 (35.6)	12.25 (4.14-36.22)	<0.001
Necrotizing pneumonia, empyema, or lung abscess	10 (8.6)	3 (6.7)	0.76 (0.20-2.89)	0.997
Management				
Infectious diseases consultation	49 (42.2)	17 (39.5)	0.87 (0.42-1.77)	0.721
Appropriate empiric antimicrobial therapy^b^	36 (31.0)	16 (35.6)	1.23 (0.59-2.53)	0.579
Vancomycin trough level ^c^				
< 15 µg/mL	45 (59.2)	11 (52.4)		
≥ 15 µg/mL	31 (40.8)	10 (47.6)	1.32 (0.50-3.48)	0.623
Mean (± SD) vancomycin level (µg/ml)^c^	14.3 (10.1)	14.5 (6.7)		0.945
MRSA PFGE type ^d^				
CMRSA-2 (USA100/800)	57 (54.8)	26 (63.4)	0.83 (0.36-1.92)	0.665
CMRSA-10 (USA300)	29 (27.9)	11 (26.8)		
Other types	18 (17.3)	4 (9.8)		
PVL gene present	30 (28.8)	11 (26.8)	0.90 (0.40-2.04)	0.998
Vancomycin MIC (µg/mL)^e^				
≤ 1.0	52 (50.0)	13 (31.7)		
≥ 1.5	52 (50.0)	28 (68.4)	2.15 (1.01-4.61)	0.053

a HCAP, healthcare-associated pneumonia; HAP, hospital-acquired pneumonia; CAP, community-acquired pneumonia

b Appropriate empiric antimicrobial therapy, treatment with vancomycin, linezolid, or clindamycin if the MRSA isolate was susceptible, in the 24-48 hrs prior to the availability of culture results.

c Vancomycin trough levels, among those treated with vancomycin and for whom vancomycin levels were available.

d PFGE, pulsed-field gel electrophoresis (145 isolates available for molecular typing)

e Vancomycin MIC, minimal inhibitory concentration (µg/mL) as determined by Etest (145 isolates available for susceptibility testing)

In the multivariable analysis the only variables that were associated with mortality were the presence of multiorgan failure (OR 8.1, 95% CI 2.5-26.0; *P*<0.001), and infection caused by MRSA isolates with reduced susceptibility to vancomcyin (MIC 1.5-2.0 µg/mL) (OR 2.5, 95% CI 1.0-6.3; *P*=0.05) (Table 4).

**Table 4:** Multivariate analysis of variables associated with 30-day all-cause mortality in patient with methicillin-resistant *Staphylococcus aureus* (MRSA) pneumonia.

**Variables**	**Adjusted Odds Ratio (95% Confidence Interval**)	***P* value**
Age (yrs)	1.03 (1.00-1.06)	0.071
Underlying cardiac or chronic pulmonary disease	0.94 (0.39-2.26)	0.886
Underlying chronic renal disease	1.61 (0.47-5.46)	0.449
CAP (community-acquired pneumonia)	0.68 (0.22-2.11)	0.506
MRSA bacteremia	2.25 (0.87-5.80)	0.094
Presence of multiorgan failure	8.09 (2.51-26.04)	< 0.001
Appropriate empiric antimicrobial therapy^a^	1.52 (0.61-3.78)	0.373
MRSA isolate with PVL gene	1.82 (0.57-5.80)	0.312
MRSA with vancomycin MIC ≥ 1.5 µg/mL^b^	2.50 (1.00-6.28)	0.051

a Appropriate empiric antimicrobial therapy, treatment with vancomycin, linezolid, or clindamycin if the MRSA isolate was susceptible, in the 24-48 hrs prior to the availability of culture results.

b Vancomycin MIC, minimal inhibitory concentration (µg/mL) as determined by Etest

## Discussion

In the past decade a decrease in the incidence of invasive healthcare-associated MRSA infections attributed to the implementation of effective infection prevention and control measures has been observed in many countries [26,27]. However, the burden of respiratory infections due to MRSA remains substantial. Approximately 36,540 pneumonias attributed to MRSA were estimated to have occurred in US hospitals in 2005 [28], and MRSA remains among the most frequently identified pathogens associated with nosocomial respiratory tract infections. However, there is considerable geographic variability in reported MRSA infection rates and assessment of the burden of disease associated with MRSA pneumonia has been difficult. Most investigations have been single center studies, and incidence has infrequently been determined. The results of this study indicate that MRSA HAP and VAP rates in Canadian hospitals are lower than those reported from a number of other countries. The incidence of MRSA HAP in French ICUs ranged from 0.50-0.90 per 1,000 patient-days [29], and mean VAP rates in US medical/surgical ICUs participating in surveillance conducted by the National Healthcare Safety Network were 0.47-0.59 per 1,000 ventilator-days [30]. A little more than one-quarter (28%) of all the pneumonias identified in our study were thought to have been community-acquired. However, MRSA appears to be an uncommon cause of CAP overall, accounting for just a small percentage of cases requiring hospital admission [3,31]. Our study was not population-based, so we were unable to determine the incidence of MRSA CAP, but estimated rates have ranged from 0.51-0.64 per 100,000 population [32].

CMRSA-10/USA300 strains represented the second most common clone identified in our study, occurring in 27.6% of cases. Most of the infections associated with CA-MRSA strains were CAP (infrequently post-influenza), but these strains were also identified in nearly 20% of patients with HAP and HCAP. These results are similar to those recently reported in a study of isolates obtained in a large international clinical trial involving patients with MRSA pneumonia [33]. Most (56.0%) of the isolates obtained globally were representative of traditional healthcare-associated strains, but the second most common (23.3%) clone was CC8, corresponding to CA-MRSA strains with SCC_*mec*_ type IV; in the US most of these isolates were USA300 strains, PVL-positive.

The 30-day all-cause mortality of 28.0% in this study is within the range (16% to 37%) of mortality rates reported in other investigations [8,9,11,34]. The role of microbial, host, and treatment variables on patient outcomes remains uncertain. In a number of studies the only independent risk factors for mortality in patients with MRSA pneumonia included host factors such as older age, and the presence of underlying chronic pulmonary disease [9,10,25]. Markers of disease severity, such as the Acute Physiology and Chronic Health Evaluation II (APACHE II) score, or requirement for vasopressor administration have also been associated with higher mortality rates [9,25]. In our study, we were unable to document APACHE II scores, but the presence of multiorgan failure, a marker for severity of disease, was the variable most strongly associated with increased risk of dying. Although we found a trend for increased mortality with increasing age, the association was not statistically significant.

The PVL gene is commonly found in CA-MRSA strains, and it has been associated with severe necrotizing *S. aureus* pneumonia with increased mortality [35,36]. However, our results are similar to those of other recent studies of MRSA HAP that did not find an association between the presence of PVL genes and increased mortality or higher risk of treatment failure [9,13,14]. A recent systematic review also found no evidence of an association between strains with PVL genes and severity of staphylococcal pneumonia [37].

Infection caused by isolates with reduced susceptibility to vancomycin has been associated with increased mortality in patients with HCAP, HAP or VAP caused by MRSA [9,11,12,38]. In our study, pneumonia caused by an isolate with a vancomycin MIC ≥ 1.5 µg/mL (as determined by Etest) was associated with increased mortality. We were not able to determine whether there were any heteroresistant vancomycin-intermediate (hVISA) isolates, but this phenotype was not associated with increased mortality in two previous studies [11,15].

Almost all patients with MRSA pneumonia in this study were treated with vancomycin, so it was not possible to assess the effect of specific antimicrobial agents on outcome. Only about one-third of patients received either vancomycin or linezolid as empiric therapy, presumably because MRSA is perceived to be a relatively uncommon cause of pneumonia in hospitalized patients in Canada. The delay in starting appropriate therapy was not associated with increased mortality, similar to findings in a study of patients with nosocomial bacteremic staphylococcal pneumonia [39], although these studies have limited power to detect a clinically significant effect. Treatment guidelines have recommended aiming for higher vancomycin trough levels (15-20 µg/mL) when this drug is being used to treat serious MRSA infections such as pneumonia [20]. However the benefit of higher vancomycin dosing has not been documented. In this study, we did not find an association between higher vancomycin trough levels and improved outcome, similar to results reported in two other investigations [25,34].

This study is the first to describe the incidence and epidemiology of MRSA pneumonia in Canadian hospitals, and was also able to identify certain host and microbial characteristics associated with outcome. However, a number of study limitations should be noted. Although we used standardized case definitions, the diagnosis of pneumonia was based on clinical criteria, and was not based on quantitative bronchoalveolar lavage cultures. As a result, cases of pneumonia may have been overdiagnosed, and patients colonized but not infected with MRSA may have been included. There may have been a survivor bias for patients with CAP, as it is possible that some patients with MRSA CAP may have died prior to hospital admission. We believe this is unlikely to have had a major impact on our results because death prior to hospitalization in patients with pneumonia in Canada is thought to occur rarely (unpublished data, Ontario Ministry of Health and Long Term Care). We also performed a left truncated survival analysis, and a logistic regression analysis stratified by whether the infection was healthcare-associated or not (data not shown), and the results of these analyses did not substantially affect the results. Although an effort was made to account for important confounders in the analysis of variables associated with mortality, important confounding covariates or interactions may have been missed. We were unable to measure severity of infection by determining clinical pulmonary infection scores (CPIS), but relied on the presence of mutiorgan failure as a clinical marker of severity of disease. The analysis of vancomycin trough levels included only the first levels obtained, and may not have reflected adjustments made to optimize vancomycin dosing. The study included a convenience sample of hospitals, predominantly teaching hospitals, and may not be representative of other healthcare facilities.

In conclusion, MRSA pneumonia rates in Canadian hospitals are relatively low, but the infection is associated with significant morbidity and 30-day mortality (28.0%). Variables independently associated with mortality were the presence of multiorgan failure, and infection caused by an isolate with reduced susceptibility to vancomycin. Additional studies are required to better understand the interaction of host factors, microbial virulence, and the impact of treatment variables on outcome in patients with MRSA pneumonia. 
